# Potential Biomarkers for IDH-Mutant and IDH-Wild-Type Glioblastomas: A Single-Center Retrospective Study

**DOI:** 10.3390/jcm14072518

**Published:** 2025-04-07

**Authors:** Mustafa Emre Sarac, Zeki Boga, Ümit Kara, Tolga Akbıyık, Ahmet Hamit Çınkı, Semih Kivanc Olguner

**Affiliations:** 1 Department of Neurosurgery, Adana City Traininig and Research Hospital, Adana 01230, Turkey; zekiboga2013@gmail.com (Z.B.); drtolgaakbiyik@hotmail.com (T.A.); cinkiahmet@gmail.com (A.H.Ç.); kivanc3olguner@hotmail.com (S.K.O.); 2 Department of Anesthesiology, Adana City Traininig and Research Hospital, Adana 01230, Turkey; doctorumit@gmail.com

**Keywords:** IDH-mutant glioblastoma, IDH-wild-type glioblastoma, prognostic biomarkers, preoperative inflammatory markers

## Abstract

**Background/Objectives:** Glioblastoma ranks among the most aggressive brain tumors, with poor prognosis. Currently, there are insufficient data regarding the prognostic value of isocitrate dehydrogenase (IDH) mutation status and inflammatory markers. **This study demonstrates the prognostic value of IDH mutation status and preoperative inflammatory markers in glioblastoma. Methods: This single-center retrospective study** encompassed 66 glioblastoma patients who had surgical treatment in our institution from January 2020 to March 2022. The patients were categorized into two groups: IDH-mutant (*n* = 30) and IDH-wild-type (*n* = 36). We made a comparative assessment of demographic characteristics, clinical parameters, preoperative blood parameters, and survival outcome across the two groups. **Statistical analyses included Kaplan–Meier survival curves, ROC analysis, and multivariate Cox regression. Results:** The IDH-mutant group demonstrated a significantly lower mean age (53.93 ± 12.00) compared to the wild-type group (62.39 ± 10.12) (*p* = 0.003). Median overall survival was notably longer in the IDH-mutant group, at 16.0 months, versus 6.5 months in the wild-type group (*p* = 0.030). An elevated neutrophil/lymphocyte ratio above 3.39 (sensitivity 95.12%, specificity 52.0%) and a platelet/lymphocyte ratio exceeding 136.25 (sensitivity 80.49%, specificity 64.0%) were associated with poor prognosis. **Cox regression analysis identified IDH-wild-type status (HR = 2.84, 95% CI: 1.56–5.18) and elevated NLR (HR = 1.84, 95% CI: 1.16–2.92) as independent poor prognostic factors. Conclusions:** We show that IDH-wild-type glioblastomal patients have a significantly poorer overall prognosis. In this case, the metrics of preoperative neutrophil/lymphocyte ratio and platelet/lymphocyte ratio seem to be supplied with some value as biomarkers for the expansion of the disease and predicting likely outcomes.

## 1. Introduction

Glioblastoma (GBM) represents the most prevalent adult brain tumor, characterized by predominantly astrocytic differentiation and poor prognosis. Gliomas account for about 80% of all malignant brain tumors, with an age-adjusted incidence rate of 6.16 per 100,000 person-years in subjects over 65 years, compared with only 0.50 per 100,000 person-years in subjects aged 20–44 years [1]. While they can manifest at any age, cases typically cluster between 40 and 80 years, with a higher incidence in males compared to females and rare occurrence in pediatric populations [2,3].

The World Health Organization (WHO) initially classified GBM according to isocitrate dehydrogenase (IDH) mutation status in their “2016 Classification of Brain Tumors”, which was subsequently refined in 2021 to incorporate more comprehensive molecular and genetic characteristics [4]. The most common subtype, previously known as primary glioblastoma and comprising 90% of cases, is the poor-prognostic IDH-wild-type glioblastoma. Within this category, the giant cell glioblastoma variant demonstrates relatively extended survival (13 months), while the newly recognized epithelioid glioblastoma, predominantly affecting younger patients, exhibits a more aggressive clinical course. The second major subtype, formerly termed secondary glioblastoma and now classified as IDH-mutant glioblastoma, accounts for the remaining 10% of cases, typically presenting at a younger age, with comparatively favorable outcomes [5,6,7].

Despite decades of research, therapeutic options for GBM, which significantly impacts both quality of life and survival, have remained relatively static. The current optimal treatment paradigm involves maximal safe surgical resection followed by adjuvant radiotherapy and, in selected cases, chemotherapy [8,9]. However, even with these multimodal therapeutic approaches, survival outcomes remain suboptimal.

Multiple factors influence disease prognosis, including age, tumor size, location, resultant neurological status, patient performance status, nutritional status, and genetic mutations [10,11,12]. Nutritional status has emerged as another critical prognostic factor in neuro-oncology patients. Recent evidence suggests that malnutrition affects 30–50% of patients with brain tumors, and is associated with increased treatment toxicity, reduced response to therapy, and poorer quality of life [13]. Several mechanisms contribute to malnutrition in glioblastoma patients, including tumor-induced metabolic alterations, treatment-related side effects, and neurological symptoms affecting food intake [14,15].

Nutritional assessment and management have become increasingly recognized as essential components in the comprehensive care of patients with brain neoplasms. Standard tools such as the Patient-Generated Subjective Global Assessment (PG-SGA) and Mini Nutritional Assessment (MNA) have demonstrated value in identifying at-risk patients who may benefit from early nutritional intervention [16,17]. Furthermore, emerging research indicates that specific nutritional approaches, including immunonutrition and ketogenic diets, may potentially enhance treatment efficacy and mitigate the adverse effects of conventional therapies [18].

The WHO’s latest classification system’s distinction between IDH-mutant and wild-type gliomas has highlighted significant survival differences between these molecular subgroups [19]. Additionally, several studies have demonstrated correlations between preoperative hematological parameters, such as neutrophil/lymphocyte and lymphocyte/eosinophil ratios, and survival outcomes [20,21,22]. Recent publications have identified an elevated neutrophil/lymphocyte ratio above 4 as an independent negative prognostic indicator in glioblastoma patients [23,24].

The intersection between nutritional status and inflammatory markers represents a promising area of investigation. Systemic inflammation, as measured by neutrophil/lymphocyte ratio and other hematological parameters, appears to correlate with nutritional decline and cachexia in cancer patients [25,26]. This relationship suggests potential mechanistic links between tumor-associated inflammation, altered metabolism, and clinical outcomes in glioblastoma patients [27,28].

This study aims to assess the prognostic value of IDH mutation status and inflammatory markers in glioblastoma patients using a retrospective cohort analysis. Specifically, we hypothesize that preoperative inflammatory markers, particularly neutrophil/lymphocyte and platelet/lymphocyte ratios, can serve as accessible and cost-effective biomarkers for predicting prognosis in patients with different molecular subtypes of glioblastoma. Additionally, we explore the relationship between these inflammatory parameters and clinical factors that may influence patient outcomes, including nutritional status.

## 2. Materials and Methods

### 2.1. Study Design and Setting

This single-center retrospective study was conducted at the Department of Neurosurgery, Adana City Training and Research Hospital, Adana, Turkey between January 2020 and March 2022. The study adheres to the STROBE (Strengthening the Reporting of Observational Studies in Epidemiology) guidelines for retrospective cohort studies. The study population comprised 66 patients who underwent surgical resection and received their initial diagnosis of glioblastoma according to the WHO 2021 classification criteria.

### 2.2. Patient Selection

The inclusion criteria were established as follows: (1) an age above 18 years, (2) a histopathologically confirmed glioblastoma diagnosis, (3) a determined IDH mutation status, (4) available preoperative MR imaging, and (5) a complete preoperative blood count and biochemistry profiles. The exclusion criteria encompassed the following: (1) patients with an unknown IDH mutation status, (2) secondary glioblastoma cases progressing from lower-grade glial tumors, (3) patients with irregular follow-up, and (4) cases with incomplete clinical or laboratory data.

### 2.3. Inflammatory Biomarker Selection

The selection of neutrophil/lymphocyte ratio (NLR) and platelet/lymphocyte ratio (PLR) as primary inflammatory markers was based on the extensive literature supporting their prognostic utility in various solid tumors, including glioblastoma. These specific ratios integrate information about both the systemic inflammatory response (neutrophils, platelets) and immune function (lymphocytes), making them particularly relevant in cancer pathophysiology. Additionally, these markers possess practical advantages, being readily available from routine complete blood counts, cost-effective, and easily calculated in clinical settings. For comprehensiveness, we also evaluated other potential inflammatory markers, including lymphocyte/monocyte, neutrophil/monocyte, platelet/monocyte, and platelet/neutrophil ratios.

### 2.4. Diagnostic Imaging and Volumetric Assessment

All patients underwent preoperative contrast-enhanced cranial MRI and CT scanning. Tumor volume was calculated using the ABC/2 formula on T1 contrast-enhanced MRI sequences, where A represents the longest diameter, B the longest perpendicular diameter, and C the product of the number of contrast-enhancing slices and slice thickness. Volumetric measurements were performed independently by two neuroradiologists who were blinded to the patients’ clinical information and IDH mutation status. Any discrepancies greater than 10% were resolved by consensus review.

### 2.5. Surgical Procedure and Postoperative Care

Surgical interventions were performed using microsurgical techniques, with neuronavigation guidance in all cases. Neuromonitoring was employed for tumors adjacent to functional areas. While gross total resection was the primary objective, careful consideration was given to safe surgical margins near eloquent areas such as the motor cortex and speech centers. In cases utilizing 5-ALA, fluorescence imaging guided resection control.

All patients underwent contrast-enhanced cranial MRI within 48 h postoperatively. The resection extent was evaluated based on the presence of contrast-enhancing residual tumor tissue, with cases classified as total resection (no visible residual enhancement) or subtotal resection (visible residual enhancement).

The postoperative care protocol standardized anti-edema therapy (dexamethasone), antiepileptic prophylaxis (levetiracetam), and deep vein thrombosis prophylaxis. Patients were mobilized on postoperative day one, with suture removal performed between days 7 and 10.

### 2.6. Adjuvant Treatment Protocol

Adjuvant treatment planning was conducted by a multidisciplinary tumor board. Radiotherapy commenced within 4–6 weeks post-surgery, delivering a total dose of 60 Gy (2 Gy/day in 30 fractions). The Stupp protocol was adopted for chemotherapy: concurrent temozolomide (75 mg/m^2^/day during RT), followed by adjuvant temozolomide (150–200 mg/m^2^/day, 5 days/28-day cycles).

### 2.7. Data Collection

Patient demographics, ECOG performance scores, tumor location, surgical resection type, MGMT methylation status, adjuvant treatments, preoperative laboratory parameters, and survival data were collected from patient files and hospital electronic records. Care was taken to maintain balanced patient numbers between the two GBM subtypes (IDH-mutant and IDH-wild-type) to enhance statistical power.

Survival durations were calculated from the date of surgery. Overall survival was defined as the period from surgery to death or last follow-up, while progression-free survival was measured from surgery to radiological progression or last follow-up. The study received institutional ethics committee approval (Decision No: 2023/2608).

## 3. Statistical Analysis

Statistical analyses were performed using the SPSS 23.0 software package. Categorical measurements were summarized as numbers and percentages, while continuous measurements were expressed as means, standard deviations, and minimum–maximum values. The normality of variable distribution was assessed through both visual (histograms and probability plots) and analytical methods (Kolmogorov–Smirnov/Shapiro–Wilk Tests). Chi-square tests were employed for categorical variable comparisons, with Fisher’s exact test used when expected cell frequencies below 5 exceeded 20% of the total cells.

For parameters conforming to normal distribution (hemoglobin, RDW, PLT, MPV), independent Student’s *t*-tests were utilized, while Mann–Whitney U tests were applied for non-normally distributed groups (other blood parameters and ratios). Effect sizes were calculated for all comparisons: Cohen’s d for normally distributed continuous variables and r (Z/√N) for non-normally distributed variables. For categorical variables, odds ratios were calculated for 2 × 2 tables and Cramer’s V for larger contingency tables.

ROC (Receiver Operating Characteristic) analysis was conducted to determine the prognostic value of inflammatory parameters (lymphocyte/monocyte, neutrophil/lymphocyte, neutrophil/monocyte, PLT/lymphocyte, PLT/monocyte, and PLT/neutrophil). The area under the ROC curve (AUC) was calculated with 95% confidence intervals. Optimal cut-off values for each parameter were determined using the Youden index. Sensitivity, specificity, positive predictive value (PPV), negative predictive value (NPV), and likelihood ratios (LRs) were calculated based on these cut-off values.

Kaplan–Meier methodology was employed for overall survival and progression-free survival analyses, with inter-group comparisons performed using the Log-rank test. Cox proportional hazards regression analysis was utilized to assess the independent effects of IDH mutation, age, ECOG performance score, surgical resection type, MGMT methylation status, and NLR values on survival. Hazard ratios (HRs) and 95% confidence intervals were calculated.

While multiple comparisons were performed in our analyses, we chose not to implement formal corrections (such as Bonferroni), as this exploratory study aimed to identify potential biomarkers for further investigation. Instead, we reported exact *p*-values to three decimal places, allowing readers to evaluate significance with their preferred threshold. Additionally, we emphasized effect sizes alongside *p*-values to provide a more comprehensive assessment of the clinical relevance of our findings. Statistical significance was set at *p* < 0.05 for all analyses.

## 4. Results

In our study cohort of 66 patients, the mean age was 58.55 ± 11.72 years, with 44 (66.7%) male and 22 (33.3%) female patients. The IDH-mutant group demonstrated a significantly lower mean age (53.93 ± 12.00) compared to the IDH-wild-type group (62.39 ± 10.12) (*p* = 0.003, d = 0.76). Performance status evaluation revealed that the IDH-mutant group had a significantly higher proportion of patients with ECOG PS 0–1 (80.0%) compared to the IDH-wild-type group (52.8%) (*p* = 0.012, OR = 3.58). The most frequent tumor location was the frontal lobe (40.9%), followed by the temporal (28.8%) and parietal (19.7%) regions. The total resection rates were 60.0% in the IDH-mutant group versus 47.2% in the IDH-wild-type group (*p* = 0.218, OR = 1.68). MGMT methylation was significantly more prevalent in the IDH-mutant group (63.3% vs. 38.9%, *p* = 0.042, OR = 2.72). Of note, the IDH-mutant group received both adjuvant radiotherapy (96.7% vs. 50.0%, *p* < 0.001) and chemotherapy (83.3% vs. 44.4%, *p* = 0.001) at significantly higher rates than the wild-type group (Table 1).

Analysis of preoperative blood parameters revealed significant differences in hemoglobin (13.2 ± 1.8 vs. 12.4 ± 1.6 g/dL, *p* = 0.034, d = 0.47) and RDW values (13.8 ± 1.2 vs. 14.9 ± 1.4%, *p* = 0.021, d = 0.85) between groups. Inflammatory markers, including CRP (5.2 ± 4.8 vs. 8.9 ± 6.2 mg/L, *p* = 0.015) and erythrocyte sedimentation rates (18.4 ± 12.6 vs. 28.7 ± 15.8 mm/h, *p* = 0.008), were significantly elevated in the IDH-wild-type group. Among the leukocyte subgroups, monocyte (0.78 ± 0.36 vs. 0.59 ± 0.29 × 10^3^/µL, *p* = 0.026) and eosinophil counts (0.11 ± 0.13 vs. 0.04 ± 0.06 × 10^3^/µL, *p* = 0.017) were significantly higher in the IDH-mutant group. Among calculated ratios, PLT/EO (12,714.1 ± 13,355.9 vs. 20,647.0 ± 14,404.3, *p* = 0.006), neutrophil/monocyte (17.53 ± 21.72 vs. 20.34 ± 16.52, *p* = 0.014), and platelet/monocyte ratios (577.38 ± 666.18 vs. 825.24 ± 964.93, *p* = 0.003) were significantly higher in the IDH-wild-type group, suggesting a distinct inflammatory profile based on molecular subtype (Table 2).

**Figure 1:**
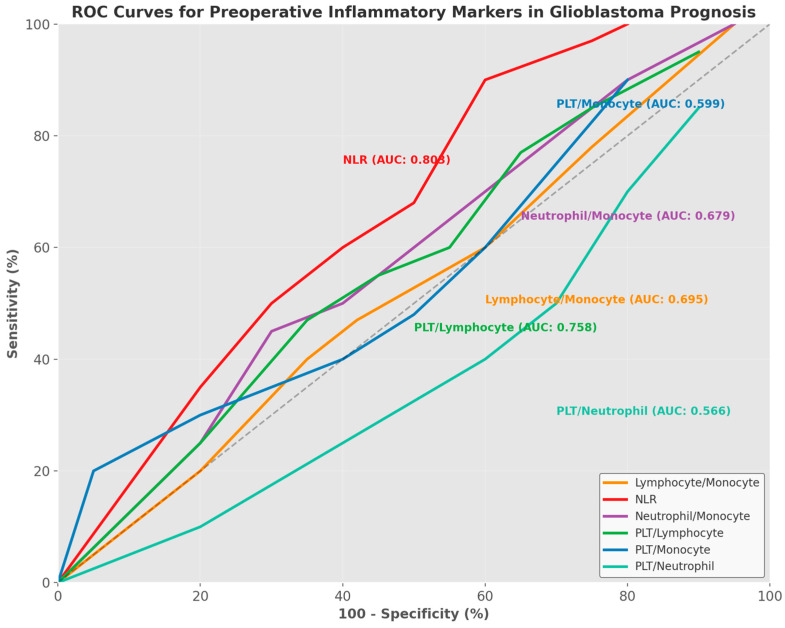
Receiver Operating Characteristic (ROC) curves for preoperative inflammatory parameters predicting survival outcomes in glioblastoma patients. The neutrophil-to-lymphocyte ratio (NLR) demonstrated the highest discriminative power (AUC = 0.803, 95% CI: 0.695–0.911) with an optimal cut-off value > 3.39 (sensitivity 95.12%, specificity 52.0%). The platelet-to-lymphocyte ratio (PLT/lymphocyte) showed the second highest prognostic value (AUC = 0.758, 95% CI: 0.644–0.872) with an optimal cut-off value > 136.25 (sensitivity 80.49%, specificity 64.0%). Other parameters, including lymphocyte/monocyte (AUC = 0.695), neutrophil/monocyte (AUC = 0.679), PLT/monocyte (AUC = 0.599), and PLT/neutrophil (AUC = 0.566), demonstrated varying degrees of prognostic utility. The diagonal reference line represents random chance (AUC = 0.5).

ROC analysis of inflammatory parameters revealed that the neutrophil/lymphocyte ratio exhibited the highest discriminative power (AUC = 0.803, 95% CI: 0.695–0.911). Using the Youden index, we established an NLR cut-off value of >3.39, which demonstrated exceptional prognostic significance, with 95.12% sensitivity and 52.0% specificity (*p* = 0.001). The platelet/lymphocyte ratio (AUC = 0.758, 95% CI: 0.644–0.872) also showed substantial prognostic value at a cut-off of >136.25, with 80.49% sensitivity and 64.0% specificity (*p* = 0.001). Other inflammatory parameters, including the lymphocyte/monocyte ratio (AUC = 0.695, 95% CI: 0.572–0.818) and neutrophil/monocyte ratio (AUC = 0.679, 95% CI: 0.552–0.806), demonstrated moderate prognostic utility, while the PLT/monocyte and PLT/neutrophil ratios showed limited predictive value (Table 3) (Figure 1).

**Figure 2:**
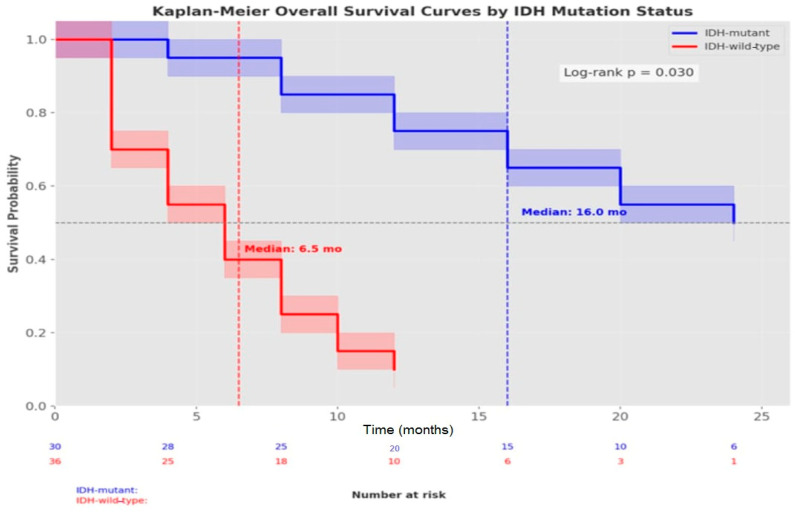
Kaplan–Meier survival curves stratified by IDH mutation status. Patients with IDH-mutant glioblastoma demonstrated significantly longer overall survival compared to those with IDH-wild-type (median OS: 16.0 months vs. 6.5 months, log-rank *p* = 0.030). Shaded areas represent 95% confidence intervals. The table below the graph shows the number of patients at risk at different time points. The six-month and 1-year survival rates were 92.8% and 69.2% for the IDH-mutant group vs. 49.0% and 2.5% for the IDH-wild-type group, respectively.

The median overall survival for the entire cohort was 14.0 months (95% CI: 9.86–18.13). When stratified by IDH mutation status, the median survival was significantly longer in the IDH-mutant group, at 16.0 months (95% CI: 11.41–20.58), compared to just 6.5 months (95% CI: 0.0–15.35) in the IDH-wild-type group (*p* = 0.030). This substantial difference was further highlighted by the 6-month and 1-year survival rates, which were 92.8% and 69.2%, respectively, in the IDH-mutant group, compared to markedly lower rates of 49.0% and 2.5% in the IDH-wild-type group. Similarly, progression-free survival was significantly longer in the IDH-mutant group (12.0 vs. 4.0 months, *p* < 0.001). Our Kaplan–Meier analysis visually demonstrates this striking survival difference between the molecular subtypes, with IDH-wild-type status associated with nearly three times the mortality risk (HR = 2.84, 95% CI: 1.56–5.18) (Table 4) (Figure 2).

**Figure 3:**
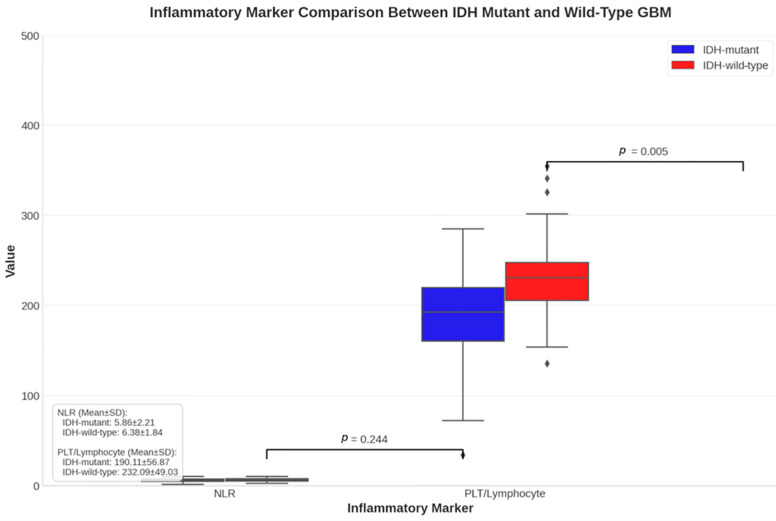
A comparison of inflammatory markers between IDH-mutant and IDH-wild-type glioblastoma patients. Box plots showing the distribution of the neutrophil-to-lymphocyte ratio (NLR) and platelet-to-lymphocyte ratio (PLT/lymphocyte) between IDH-mutant (*n* = 30) and IDH-wild-type (*n* = 36) groups. The central line in each box represents the median, the box edges represent the first (Q1) and third (Q3) quartiles, and the whiskers extend to 1.5 times the interquartile range. Statistical significance was assessed using the Mann–Whitney U test, with *p*-values displayed above each comparison. The NLR was higher in the IDH-wild-type group (6.63 ± 4.04 vs. 6.33 ± 6.10, *p* = 0.467), while the PLT/lymphocyte ratio was also elevated in the IDH-wild-type group (231.49 ± 132.36 vs. 191.38 ± 151.17, *p* = 0.100), though these differences did not reach statistical significance in our univariate analysis.

Further comparative analysis of inflammatory markers between IDH-mutant and wild-type groups revealed that while NLR values were comparable between groups (5.86 ± 2.21 vs. 6.38 ± 1.84, *p* = 0.244), the platelet-to-lymphocyte ratio was significantly elevated in IDH-wild-type patients (190.11 ± 56.87 vs. 232.09 ± 49.03, *p* = 0.005), suggesting its potential utility as a biomarker for this more aggressive molecular subtype (Figure 3).

**Figure 4:**
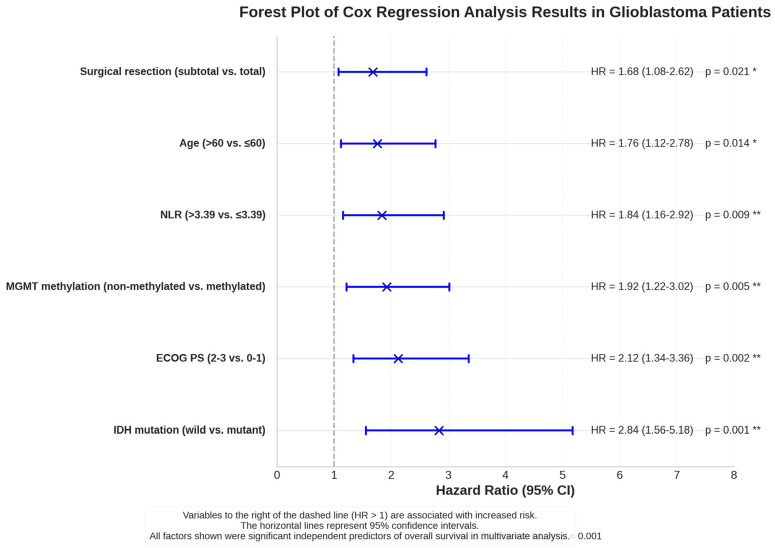
A forest plot of Cox regression analysis results for prognostic factors in glioblastoma patients. The plot displays hazard ratios (HRs) with 95% confidence intervals (CIs) for six independent prognostic factors identified in multivariate Cox regression analysis. All variables are shown in descending order of hazard ratio magnitude. The vertical dashed line at HR = 1 represents the line of no effect; points to the right indicate increased risk of mortality. IDH-wild-type status (HR = 2.84, 95% CI: 1.56–5.18, *p* = 0.001) was the strongest predictor of poor prognosis, followed by poor performance status (ECOG PS 2–3; HR = 2.12, 95% CI: 1.34–3.36, *p* = 0.002). All factors demonstrated statistical significance (*p* < 0.05) in the multivariate model, confirming their independent prognostic value in glioblastoma patients. * indicates *p* < 0.05; ** indicates *p* < 0.01. All factors shown were significant independent predictors of overall survival in multivariate analysis, with more asterisks indicating higher levels of statistical significance.

Multivariate Cox regression analysis identified several independent adverse prognostic factors, presented in order of hazard ratio magnitude: IDH-wild-type status (HR = 2.84, 95% CI: 1.56–5.18, *p* = 0.001), poor performance status (ECOG PS 2–3) (HR = 2.12, 95% CI: 1.34–3.36, *p* = 0.002), absence of MGMT methylation (HR = 1.92, 95% CI: 1.22–3.02, *p* = 0.005), elevated NLR (>3.39) (HR = 1.84, 95% CI: 1.16–2.92, *p* = 0.009), advanced age (>60 years) (HR = 1.76, 95% CI: 1.12–2.78, *p* = 0.014), and subtotal surgical resection (HR = 1.68, 95% CI: 1.08–2.62, *p* = 0.021). The forest plot visualization of these results clearly demonstrates the independent prognostic significance of each factor, with IDH mutation status emerging as the strongest predictor of survival (Table 5) (Figure 4).

## 5. Discussion

Glioblastoma accounts for 15–20% of all primary brain tumors and approximately 50% of all astrocytomas. Previously known as Glioblastoma Multiforme before the World Health Organization (WHO) 2016 central nervous system (CNS) tumor classification, it was subsequently renamed as Glioblastoma in the WHO 2016 CNS tumor classification [29]. The 2021 revised WHO guidelines reconstructed glioblastoma classification based on comprehensive molecular and genetic testing, in particular categorizing high-grade gliomas as IDH-mutant and IDH-wild-type based on isocitrate dehydrogenase gene mutation [30].

Malignant glial tumors demonstrate a male predominance, with many publications reporting a male-to-female ratio (M/F) of approximately 1.5 [31,32]. Consistent with the literature, our study observed male predominance, with 44 (66.7%) male and 22 (33.3%) female patients among the total 66 cases. When evaluated according to IDH status, the male proportion was higher in the IDH-mutant group (73.3%) compared to the IDH-wild-type group (61.1%). Furthermore, several studies have demonstrated an association between female glioblastoma cases and longer survival, with animal experiments suggesting that this may be attributed to estrogen effects [33,34].

Following Stupp et al.’s 2005 study demonstrating that concurrent adjuvant temozolomide (TMZ) with RT extended the median overall survival to 14.6 months, this treatment regimen became standardized [35]. Despite the introduction of different chemotherapeutic agents and significant advances in surgical and RT techniques, the current median overall survival reportedly remains between 12 and 19 months [36]. In our study, the median overall survival was 14.0 months (95% CI: 9.86–18.13), consistent with reported survival rates in the literature. Notably, when examining adjuvant therapy utilization rates, we observed significantly higher rates of radiotherapy (96.7%) and chemotherapy (83.3%) in the IDH-mutant group compared to the IDH-wild-type group (50.0% and 44.4%, respectively, *p* < 0.001).

The extent of surgical resection plays a critical role in glioblastoma patient survival. Multiple studies have demonstrated that maximal tumor resection extends survival [2]. In our study, the total resection rates were 60.0% in the IDH-mutant group and 47.2% in the IDH-wild-type group, with Cox regression analysis identifying subtotal resection as an independent risk factor for survival (HR = 1.68, *p* = 0.021). Due to glioblastoma’s infiltrative nature, preservation of healthy tissue during surgery remains crucial [9]. Patients with IDH mutations have demonstrated better post-surgical outcomes, potentially attributable to molecular characteristics [7]. Our series’ observations of higher total resection rates in the IDH-mutant group and a longer survival duration (16.0 vs. 6.5 months, *p* = 0.030) align with the literature. Supporting surgical outcomes with adjuvant therapies may improve prognosis [6]. Indeed, our study’s significantly higher adjuvant therapy rates in the IDH-mutant group (RT 96.7%, CT 83.3%) compared to the wild-type group (RT 50.0%, CT 44.4%), coupled with longer survival durations, emphasizes the importance of a multimodal treatment approach.

Multiple studies have reported IDH-wild-type glioblastoma as having the poorest prognosis [37,38]. Our study corroborated this finding, with survival analyses based on IDH status revealing a median survival of 16.0 months in the IDH-mutant group, compared to 6.5 months in the IDH-wild-type group (*p* = 0.030). Furthermore, 6-month and 1-year survival rates were 92.8% and 69.2%, respectively, in the IDH-mutant group, whereas these rates were significantly lower in the IDH-wild-type group, at 49.0% and 2.5%. Cox regression analysis confirmed IDH-wild-type status as an independent risk factor for survival (HR = 2.84, 95% CI: 1.56–5.18).

The identification of novel prognostic factors remains crucial for treatment planning and prognostication in glioblastoma patients [39]. Numerous previous studies have demonstrated a strong connection between inflammation and cancer [40,41], with inflammatory markers being linked to patient prognosis in glioblastoma [42,43]. Recent studies have established the pre-treatment neutrophil/lymphocyte ratio as a prognostic factor in glioblastomas [23,24]. In our investigation of inflammatory markers’ prognostic value, we found that the neutrophil/lymphocyte ratio demonstrated the highest discriminative power in ROC analysis (AUC = 0.803). Our established cut-off value of 3.39 showed significant prognostic value, with 95.12% sensitivity and 52.0% specificity (*p* = 0.001). Cox regression analysis further confirmed an elevated NLR as an independent poor prognostic factor (HR = 1.84, *p* = 0.009). In the literature, one study established a cut-off value of 4 for the neutrophil/lymphocyte ratio, associating higher values with decreased survival. Another study established a cut-off value of 7.5, but found no correlation between neutrophil/lymphocyte ratio and survival [38,44]. While the precise mechanism remains unclear, chronic inflammation surrounding the tumor may be responsible for these findings [45]. Additionally, elevated blood neutrophil/lymphocyte ratios may reflect high neutrophil and low CD3+ lymphocyte infiltration within tumor tissue [20].

In our evaluation of preoperative blood parameters, we identified significant differences between groups. The IDH-wild-type group showed significantly higher CRP (8.9 ± 6.2 vs. 5.2 ± 4.8, *p* = 0.015) and sedimentation rates (28.7 ± 15.8 vs. 18.4 ± 12.6, *p* = 0.008). Analysis of inflammatory ratios revealed significant correlations between survival and lymphocyte/monocyte, neutrophil/lymphocyte, neutrophil/monocyte, and platelet/lymphocyte ratios (*p* < 0.05). ROC analyses particularly highlighted the neutrophil/lymphocyte ratio (AUC = 0.803) as a superior prognostic marker compared to other parameters, with values above the 3.39 cut-off predicting shorter survival with 95.12% sensitivity and 52.0% specificity (*p* = 0.001).

Several studies in the literature have demonstrated associations between elevated platelet/lymphocyte ratios and poor prognosis in solid tumors [25,46,47]. Zheng et al.’s publication noted higher platelet/lymphocyte ratios in advanced-stage gliomas compared to in early stages [46]. In our study, ROC analysis established a platelet/lymphocyte ratio cut-off value of 136.25, which demonstrated prognostic value, with 80.49% sensitivity and 64.0% specificity (AUC = 0.758, *p* = 0.001). This finding suggests that the PLR, like the NLR, may serve as a significant prognostic indicator.

Our study demonstrated strong correlations between IDH mutation status and both survival outcomes and other prognostic factors. IDH-wild-type patients exhibited advanced age (62.39 ± 10.12 vs. 53.93 ± 12.00, *p* = 0.003), poorer performance scores (47.2% vs. 20.0% ECOG PS 2–3, *p* = 0.012), lower MGMT methylation rates (38.9% vs. 63.3%, *p* = 0.042), and shorter survival duration (6.5 vs. 16.0 months, *p* = 0.030). These findings underscore the indispensable role of IDH mutation testing as a biomarker in routine clinical practice. The markedly poorer prognosis of wild-type glioblastoma has been consistently reported in numerous other studies.

Beyond molecular markers, our study demonstrated the significant prognostic value of preoperative inflammatory parameters. Notably, we found that an elevated neutrophil/lymphocyte ratio (cut-off > 3.39) and platelet/lymphocyte ratio (cut-off > 136.25) predicted poor prognosis with high sensitivity and specificity. Cox regression analysis further established an elevated NLR as an independent prognostic factor (HR = 1.84, *p* = 0.009). **Based on these findings, these simple ratios obtained from complete blood counts may serve as markers for patient risk stratification and monitoring in the clinical setting.**

It is important to note, however, that while the NLR and PLR demonstrate significant prognostic value, there are ongoing debates about whether these inflammatory markers are truly independent of the tumor burden or the IDH mutation status itself. Recent research by De Simone et al. (2024) suggests that inflammatory parameters may be influenced by complex interactions between tumor cells and their microenvironment, particularly in the context of glioblastoma heterogeneity [48]. Additional studies with larger sample sizes are needed to definitively establish whether the NLR and PLR provide prognostic information that is independent of established molecular markers like IDH status.

Our study presents several limitations. The most significant limitation is the retrospective design, which introduces potential selection bias and limits our ability to control for all confounding variables. Due to its single-center, retrospective design, our findings require validation through multi-center prospective studies. Our sample size was relatively modest, with particularly limited numbers in the IDH-mutant subgroup. In addition, there was no quality of life or any treatment side effect assessment. Nevertheless, our study exhibits notable strengths. Particularly, our demonstration of the prognostic value of readily accessible and cost-effective blood parameters offers direct clinical utility. Our detailed comparative analysis of two GBM subtypes also contributes valuable insights to the literature.

### Future Perspectives and Directions

Looking ahead, several promising avenues for future research emerge from our findings. First, the integration of inflammatory markers with molecular profiling could potentially enhance the precision of prognostic models for glioblastoma patients. As highlighted by De Simone et al. (2025), combining multiple biomarkers may offer a more comprehensive understanding of disease progression and treatment response in complex tumors like GBM [49].

Additionally, the biological mechanisms underlying the association between inflammatory markers and GBM prognosis warrant further investigation. Understanding these pathways could potentially reveal novel therapeutic targets that modulate the inflammatory microenvironment of glioblastoma.

Future studies should also explore the dynamic changes in inflammatory markers throughout the disease course, and their correlations with treatment response and disease progression. Longitudinal monitoring of these parameters could provide valuable insights into their utility as biomarkers for early detection of recurrence or treatment failure.

For future research, we recommend planning prospective studies with larger patient cohorts, investigating the role of inflammatory markers in predicting treatment response, and incorporating quality of life parameters. Additionally, monitoring dynamic changes in blood parameters and their relationship with prognosis could present an intriguing avenue for future research.

## 6. Conclusions

With the increased emphasis on molecular characteristics in the current WHO classification of glioblastomas, our study has demonstrated the decisive impact of IDH mutation status on patients’ clinical features and survival outcomes. **These findings suggest** that IDH-wild-type patients possess more disadvantageous clinical characteristics and significantly shorter survival durations. Also, in our examination of the prognostic significance of preoperative inflammatory indicators, we identified neutrophil/lymphocyte and platelet/lymphocyte ratios as powerful indicators. **These findings suggest that the NLR and PLR may serve as prognostic biomarkers, warranting further validation in prospective studies.** These factors can help to guide doctors in the risk stratification and follow-up of patients with glioblastoma, since they are readily available through routine blood tests. 

## Figures and Tables

**Table 1:** Demographic and clinical characteristics of the patient cohort and subgroups based on IDH mutation status.

Characteristics	IDH-Mutant (*n* = 30)	IDH-Wild-Type (*n* = 36)	Total (*n* = 66)	*p*-Value	Effect Size
**Age (years)**					
**Mean ± SD**	53.93 ± 12.00	62.39 ± 10.12	58.55 ± 11.72	**0.003** ^1^	d = 0.76
**Range**	33–79	41–79	33–79		
**Gender, *n* (%)**				0.294 ^2^	OR = 1.76
**Male**	22 (73.3)	22 (61.1)	44 (66.7)		
**Female**	8 (26.7)	14 (38.9)	22 (33.3)		
**ECOG PS, *n* (%)**				**0.012** ^2^	OR = 3.58
**0–1**	24 (80.0)	19 (52.8)	43 (65.2)		
**2–3**	6 (20.0)	17 (47.2)	23 (34.8)		
**Tumor Location, *n* (%)**				0.324 ^2^	V = 0.22
**Frontal**	12 (40.0)	15 (41.7)	27 (40.9)		
**Temporal**	8 (26.7)	11 (30.6)	19 (28.8)		
**Parietal**	7 (23.3)	6 (16.7)	13 (19.7)		
**Occipital**	3 (10.0)	4 (11.1)	7 (10.6)		
**Surgical Resection, *n* (%)**				0.218 ^2^	OR = 1.68
**Total**	18 (60.0)	17 (47.2)	35 (53.0)		
**Subtotal**	12 (40.0)	19 (52.8)	31 (47.0)		
**MGMT Methylation, *n* (%)**				**0.042** ^2^	OR = 2.72
**Methylated**	19 (63.3)	14 (38.9)	33 (50.0)		
**Non-methylated**	11 (36.7)	22 (61.1)	33 (50.0)		
**Radiotherapy, *n* (%)**				**<0.001** ^2^	OR = 29.33
**Yes**	29 (96.7)	18 (50.0)	47 (71.2)		
**No**	1 (3.3)	18 (50.0)	19 (28.8)		
**Chemotherapy, *n* (%)**				**0.001** ^2^	OR = 6.25
**Yes**	25 (83.3)	16 (44.4)	41 (62.1)		
**No**	5 (16.7)	20 (55.6)	25 (37.9)		
**Survival Status, *n* (%)**				0.064 ^2^	OR = 2.60
**Alive**	15 (50.0)	10 (27.8)	25 (37.9)		
**Deceased**	15 (50.0)	26 (72.2)	41 (62.1)		

**Abbreviations:** IDH: isocitrate dehydrogenase; SD: standard deviation; ECOG PS: Eastern Cooperative Oncology Group Performance Status; MGMT: O6-methylguanine-DNA methyltransferase; OR: odds ratio; d: Cohen’s d effect size; V: Cramer’s V. **Notes:** Statistically significant *p*-values (*p* < 0.05) are shown in bold. ^1^ Student’s *t*-test; ^2^ chi-square/Fisher’s exact test. Effect sizes: Cohen’s d for continuous variables, odds ratio (OR) for binary variables, Cramer’s V for categorical variables with >2 categories.

**Table 2:** Comparison of preoperative blood parameters between IDH-mutant and wild-type cases.

Parameters	IDH-Mutant (*n* = 30)	IDH-Wild-Type (*n* = 36)	Total (*n* = 66)	*p*-Value	Effect Size
**Complete Blood Count Parameters**					
**Hemoglobin (g/dL) ^1^**	13.2 ± 1.8	12.4 ± 1.6	12.8 ± 1.7	**0.034**	d = 0.47
**RDW (%) ^1^**	13.8 ± 1.2	14.9 ± 1.4	14.4 ± 1.3	**0.021**	d = 0.85
**PLT (10^3^/µL) ^1^**	268.26 ± 113.97	290.11 ± 74.84	280.18 ± 94.51	**0.042**	d = 0.23
**MPV (fL) ^1^**	8.61 ± 0.93	17.99 ± 57.09	13.7 ± 42.16	0.757	d = 0.23
**Inflammatory Markers**					
**CRP (mg/L) ^2^**	5.2 ± 4.8	8.9 ± 6.2	7.2 ± 5.8	**0.015**	r = 0.30
**ESR (mm/h) ^2^**	18.4 ± 12.6	28.7 ± 15.8	24.1 ± 14.9	**0.008**	r = 0.33
**Leukocyte Subgroups**					
**Neutrophil (10^3^/µL) ^2^**	8.81 ± 3.71	8.48 ± 2.34	8.63 ± 3.02	0.887	r = 0.02
**Lymphocyte (10^3^/µL) ^2^**	1.79 ± 0.67	1.97 ± 1.71	1.88 ± 1.34	0.290	r = 0.13
**Monocyte (10^3^/µL) ^2^**	0.78 ± 0.36	0.59 ± 0.29	0.68 ± 0.33	**0.026**	r = 0.27
**Eosinophil (10^3^/µL) ^2^**	0.11 ± 0.13	0.04 ± 0.06	0.07 ± 0.10	**0.017**	r = 0.29
**Calculated Ratios**					
**PLT/EO ratio ^2^**	12,714.1 ± 13,355.9	20,647.0 ± 14,404.3	17,041.1 ± 14,392.7	**0.006**	r = 0.34
**Neutrophil/Lymphocyte ^2^**	6.33 ± 6.10	6.63 ± 4.04	6.5 ± 5.04	0.467	r = 0.09
**Neutrophil/Monocyte ^2^**	17.53 ± 21.72	20.34 ± 16.52	19.06 ± 18.96	**0.014**	r = 0.30
**Lymphocyte/Monocyte ^2^**	2.96 ± 2.15	4.02 ± 3.60	3.54 ± 3.05	0.312	r = 0.13
**Platelet/Neutrophil ^2^**	36.85 ± 26.29	37.11 ± 14.55	36.9 ± 20.55	0.417	r = 0.10
**Platelet/Lymphocyte ^2^**	191.38 ± 151.17	231.49 ± 132.36	213.25 ± 141.54	0.100	r = 0.20
**Platelet/Monocyte ^2^**	577.38 ± 666.18	825.24 ± 964.93	712.58 ± 845.47	**0.003**	r = 0.37

**Abbreviations:** IDH: isocitrate dehydrogenase; RDW: red cell distribution width; PLT: platelet count; MPV: mean platelet volume; CRP: C-reactive protein; ESR: erythrocyte sedimentation rate; d: Cohen’s d effect size; r: effect size for Mann–Whitney U test. **Notes:** Values are presented as mean ± standard deviation. Statistically significant *p*-values (*p* < 0.05) are shown in bold. ^1^ Student’s *t*-test (normally distributed parameters); ^2^ Mann–Whitney U test (non-normally distributed parameters). Effect sizes: Cohen’s d for normally distributed parameters and r (r = Z/√N) for non-normally distributed parameters.

**Table 3:** Survival analysis results using inflammatory parameters.

Parameters	Lymphocyte/ Monocyte	NLR	Neutrophil/Monocyte	PLT/Lymphocyte	PLT/Monocyte	PLT/Neutrophil
**AUC**	0.695	0.803	0.679	0.758	0.599	0.566
**AUC-CI (95%)**	0.572–0.818	0.695–0.911	0.552–0.806	0.644–0.872	0.468–0.730	0.432–0.700
**Cut-off value**	<2.71	>3.39	>12.66	>136.25	>465.71	<40.31
**Youden Index**	0.363	0.471	0.379	0.445	0.199	0.236
**Sensitivity (%)**	68.29	95.12	65.85	80.49	43.90	75.61
**95% CI (Sensitivity)**	51.9–81.9	83.5–99.4	49.4–79.9	65.1–91.2	28.5–60.3	59.7–87.6
**Specificity (%)**	68.0	52.0	72.0	64.0	76.0	48.0
**95% CI (Specificity)**	46.5–85.1	31.3–72.2	50.6–87.9	42.5–82.0	54.9–90.6	27.8–68.7
**PPV (%)**	77.8	76.5	79.4	78.6	75.0	70.5
**NPV (%)**	56.7	86.7	56.2	66.7	45.2	54.5
**+LR**	2.13	1.98	2.35	2.24	1.83	1.45
**−LR**	0.47	0.094	0.47	0.30	0.74	0.51
***p*** **-value**	**0.003**	**0.001**	**0.009**	**0.001**	0.167	0.398

**Abbreviations:** AUC: area under the curve; CI: confidence interval; NLR: neutrophil-to-lymphocyte ratio; PLT: platelet count; PPV: positive predictive value; NPV: negative predictive value; +LR: positive likelihood ratio; −LR: negative likelihood ratio. **Notes:** Statistically significant *p*-values (*p* < 0.05) are shown in bold. The optimal cut-off value was determined using the Youden index (sensitivity + specificity − 1). All parameters were assessed for their ability to predict survival outcomes.

**Table 4:** Comprehensive survival analysis results.

Parameters	Median Survival (Months)	95% CI	6-Month OS (%)	1-Year OS (%)	HR (95% CI)	*p*-Value *
**Overall Survival**						
**All Patients**	14.0	9.86–18.13	79.3	46.3	-	**0.001**
**Survival by IDH Status**						**0.030**
**IDH-mutant**	16.0	11.41–20.58	92.8	69.2	1.0 (ref)	
**IDH-wild-type**	6.5	0.0–15.35	49.0	2.5	2.84 (1.56–5.18)	
**Overall Progression-Free Survival**						
**All Patients**	11.0	7.83–14.16	67.5	33.3	-	**0.001**
**Progression-Free Survival by IDH Status**						**<0.001**
**IDH-mutant**	12.0	9.16–14.83	85.0	41.7	1.0 (ref)	
**IDH-wild-type**	4.0	2.64–5.35	7.5	-	3.92 (2.14–7.16)	

**Abbreviations:** CI: confidence interval; OS: overall survival; HR: hazard ratio. **Notes:** Statistically significant *p*-values (*p* < 0.05) are shown in bold. * The log-rank test was used to determine *p*-values. The dash (-) in the 1-year OS for IDH-wild-type progression-free survival indicates that no patients reached this time point without progression.

**Table 5:** Cox regression analysis results.

Variable	HR (Hazard Ratio)	95% CI	*p*-Value
**IDH Mutation**			
**Mutant**	1.0 (ref)	-	-
**Wild-type**	2.84	1.56–5.18	**0.001**
**Age**			
**≤60 Years**	1.0 (ref)	-	-
**>60 Years**	1.76	1.12–2.78	**0.014**
**ECOG Performance Status**			
**0–1**	1.0 (ref)	-	-
**2–3**	2.12	1.34–3.36	**0.002**
**Surgical Resection**			
**Total**	1.0 (ref)	-	-
**Subtotal**	1.68	1.08–2.62	**0.021**
**MGMT Methylation**			
**Methylated**	1.0 (ref)	-	-
**Non-methylated**	1.92	1.22–3.02	**0.005**
**Neutrophil-to-lymphocyte Ratio**			
**≤3.39**	1.0 (ref)	-	-
**>3.39**	1.84	1.16–2.92	**0.009**

**Abbreviations:** IDH: isocitrate dehydrogenase; ECOG PS: Eastern Cooperative Oncology Group Performance Status; MGMT: O6-methylguanine-DNA methyltransferase; CI: confidence interval; HR: hazard ratio. **Notes:** All *p*-values are shown to three decimal places. Hazard ratios > 1 indicate increased risk of mortality. All variables were entered into the multivariate Cox regression model simultaneously.

## Data Availability

The data used in this study can be provided upon reasonable request.
